# Spatiotemporal modeling of tau aggregation following traumatic brain injury: implications for chronic traumatic encephalopathy

**DOI:** 10.1007/s10237-026-02092-4

**Published:** 2026-06-05

**Authors:** José González-Cabrero, Carlos G. S. Cardoso, Inés Moreno-González, Ricardo J. Alves-de-Sousa

**Affiliations:** 1 Centre for Mechanical Technology and Automation (TEMA), Department of Mechanical Engineering, Campus Universitário de Santiago, University of Aveiro, 3810-193 Aveiro, Portugal; 2 LASI—Intelligent Systems Associate Laboratory, 4800-058 Guimarães, Portugal; 3 Department of Cell Biology, Genetics and Physiology, Instituto de Investigacion Biomedica de Malaga - IBIMA-Plataforma Bionand, Faculty of Sciences, Malaga University, Malaga, Spain; 4 Centro de Investigacion Biomedica en Red Sobre Enfermedades Neurodegenerativas (CIBERNED), Madrid, Spain; 5 Department of Neurology, The University of Texas Health Science Center at Houston, Houston, TX USA

**Keywords:** Traumatic brain injury, Chronic traumatic encephalopathy, Tau aggregation, Finite element modeling, Spatiotemporal tau prediction

## Abstract

Chronic traumatic encephalopathy (CTE) is a progressive neurodegenerative disorder associated with repetitive traumatic brain injury (TBI) and characterized by abnormal tau aggregation. Although biological and clinical evidence link head trauma to tau pathology, the mechanistic pathway connecting mechanical impact to biochemical progression remains insufficiently defined. This research introduces a mathematical model designed to predict the spatiotemporal evolution of tau accumulation following TBI. The formulation is based on an Avrami-type nucleation–growth framework, originally developed for phase transformations in materials, here adapted to represent the initiation and expansion of tau aggregates. Nucleation rate and growth velocity are treated as time-dependent parameters to capture realistic pathological dynamics. Temporal kinetics are calibrated using experimental data from mouse models of tauopathy, ensuring agreement with observed global progression. Spatial heterogeneity is incorporated through a mechanical field derived from finite element simulations, enabling regions exposed to higher post-impact strain to exhibit faster local transformation. This integrated biomechanical–mathematical approach provides a quantitative link between injury-induced deformation and tau aggregation, offering a basis for identifying mechanically vulnerable regions that may be predisposed to CTE-related pathology.

## Introduction

Traumatic brain injury (TBI) is a pervasive public-health problem and a recognized antecedent of long-term neurological decline (Gardner and Yaffe 2015). Across contact sports, military blast exposure, and civilian falls or traffic accidents, repeated concussive and sub-concussive impacts have been associated with later cognitive impairment, mood disturbance, and behavioral dysfunction (Covassin et al. 2008; McInnes et al. 2017). A central neuropathological hallmark across these scenarios is the progressive accumulation of abnormally phosphorylated tau, forming neurofibrillary tangles and threads that disrupt axonal transport, synaptic communication, and the function of large-scale neural networks (Honson et al. 2008; Guo et al. 2017). Histopathological studies, such as those by McKee et al. (McKee et al. 2009; McKee et al. 2013), have documented characteristic perivascular and sulcal-depth-accentuated tau deposition in chronic traumatic encephalopathy (CTE), along with a staging scheme that progresses from focal cortical involvement to a widespread cortical and subcortical burden.

There is now substantial evidence that mechanical deformation of axons plays a direct causal role in initiating tau pathology. Rapid stretch or shear deformation disrupts microtubules, impairs axonal transport, and causes local axonal damage in the central nervous system white matter (Bain and Meaney 2000; Meaney and Smith 2011; Giordano and Kleiven 2014). Experimental models further show that TBI can induce tau aggregation and promote its spatiotemporal spreading (Iii et al. 2020), while neuronal activity enhances the trans-neuronal propagation of tau pathology (Wu et al. 2016). Computational and multiscale biomechanical studies quantify how inertial loading and local strain patterns can damage axons under head impacts (Wright et al. 2013; Zhan et al. 2025), providing a mechanistic link between macroscopic kinematics and microscopic injury. Moreover, tau pathology is known to propagate along anatomically and functionally connected pathways in both animal models and humans (Vogel et al. 2020; Calignon et al. 2012; Cornblath et al. 2021; Zheng et al. 2019; Raj et al. 2012), suggesting that mechanically compromised axonal networks provide a substrate for the subsequent spread of misfolded tau. Together, these observations support a mechanobiological cascade in which TBI-induced axonal deformation initiates tau aggregation, which then self-propagates through neural circuits and may be further modulated by repetitive head trauma and sport-related mechanical exposures (Jucker et al. 2013; Horstemeyer et al. 2019; Butler et al. 2025).

Despite these advances, a quantitative bridge between injury biomechanics and biochemical tau kinetics remains incomplete. Diffusion- and connectome-based propagation models reproduce aspects of the anatomical spread of tau, typically by coupling local vulnerability with network-mediated dissemination of pathology (Cornblath et al. 2021; Zheng et al. 2019; Raj et al. 2012). However, these models do not specify where pathological domains first arise at the microscale or how local mechanical loading sets the initial conditions for aggregation. More mechanistic mathematical models of neurodegenerative pathogenesis have been proposed for Alzheimer’s disease (Puri and Li 2010), and multiscale frameworks have been developed to relate mechanical insult to tau accumulation in specific cohorts, such as American football players (Horstemeyer et al. 2019). However, these approaches either omit explicit nucleation-and-growth kinetics or remain computationally demanding for whole-brain simulations.

A mechanistically interpretable alternative is offered by the Kolmogorov–Johnson–Mehl–Avrami (KJMA) theory of nucleation and growth processes (Fanfoni et al. 1998; Avrami 1939; Barmak and Barmak 2010). In this framework, the temporal evolution of a transformed volume percentage is governed by the rates of nucleation, deterministic growth, and impingement of growing domains, yielding characteristic sigmoidal kinetics. KJMA and related Avrami-type formalisms have been extensively analysed in materials science and beyond (Fanfoni et al. 1998; Avrami 1939; Barmak and Barmak 2010; Shirzad and Viney 2023) and successfully applied to protein-aggregation phenomena, including amyloid and polyglutamine fibril formation, as well as self-propagating pathogenic protein aggregates in neurodegenerative disease (Jucker et al. 2013). In the context of tauopathy, nucleation can be interpreted as the injury-induced formation of misfolded tau seeds, whereas radial growth models the local expansion of tau-affected microdomains until they impinge within neural tissue.

Mechanical cues are increasingly recognized as key modulators of neurodegenerative vulnerability. Experimental work has established quantitative thresholds for axonal deformation leading to cytoskeletal damage in central nervous system white matter (Bain and Meaney 2000; Meaney and Smith 2011; Giordano and Kleiven 2014). Finite-element (FE) simulations of head impact and brain deformation, informed by regional mechanical properties of human brain tissue (Menichetti et al. 2020) and detailed human head geometries (Carmo et al. 2023), consistently identify stress- and strain-concentrating anatomical regions such as sulcal depths and gray–white interfaces (Wright et al. 2013; Zhan et al. 2025). However, these mechanically derived fields have rarely been embedded within explicit biochemical transformation laws for tau aggregation.

The present work addresses this gap by incorporating FE-derived mechanical loading into an Avrami-type kinetic framework, allowing local mechanical strain to modulate both nucleation and growth rates of tau aggregation For temporal calibration, this research relies on the experimental study by Edwards et al. (2020), in which a single controlled cortical impact (CCI) was delivered to tau-transgenic mice after craniotomy, directly over one cerebral hemisphere, generating an indentation-type injury that subsequently led to progressive tau aggregation. CTE, however, is primarily associated with repetitive head impacts, while the present model is calibrated using a single-impact murine TBI dataset. Accordingly, this study should be interpreted as an exploratory analysis of one mechanobiological component of trauma-induced tau aggregation that may contribute to CTE-related pathology, not as a complete representation of the disease process. The calibrated law is then projected onto a human-scale FE mesh constructed from validated head models and subject-specific mechanical-property assignments (Carmo et al. 2023; Alshareef et al. 2021), enabling spatiotemporal predictions τ(x,t) that reflect both biological kinetics and the underlying mechanical environment. Therefore, the primary aim of this exploratory study is to introduce a mathematical framework that links mechanical outputs, such as maximum principal strain, to the spatial distribution of tau accumulation, using the murine experiment as temporal biological calibration.

This research aims to: 1) formulate a biomechanics-informed Avrami model for tau aggregation with explicit physical meanings for nucleation, growth, and saturation; 2) calibrate the temporal kinetics using experimental mouse data from Edwards et al. (Iii et al. 2020); 3) integrate the calibrated kinetics with FE-derived strain fields to generate spatiotemporal predictions τ(x, t) of tau accumulation that identify mechanically vulnerable brain regions.

## Materials and methodology

Experimental measurements from tau-transgenic mice subjected to TBI were compiled, and the temporal evolution of aggregated tau was used as the biological reference for kinetic calibration. A mathematical formulation based on the Avrami nucleation–growth framework was then established to describe tau aggregation in terms of the appearance of new pathological nuclei and the time-dependent expansion of existing aggregates. Calibration of the model parameters against the experimental data yielded the kinetic constants governing the initiation and growth of the tau protein.

In parallel, a biomechanical simulation was performed on a three-dimensional head model to characterize the mechanical response of brain tissue during a frontal impact. The simulation provided a spatial field of logarithmic maximum principal strain (MPS), used as a surrogate for mechanical deformation. After extracting the nodal MPS values from the simulation output, the field was normalized and incorporated into the kinetic formulation to introduce spatial heterogeneity, enabling regions subjected to higher deformation to exhibit faster local transformation rates. In this sense, the murine dataset is used as a biological reference to calibrate the temporal evolution of the model, whereas the human finite-element simulation is used to provide a mechanically meaningful spatial field for exploratory projection of tau distribution.

**Fig. 1:**
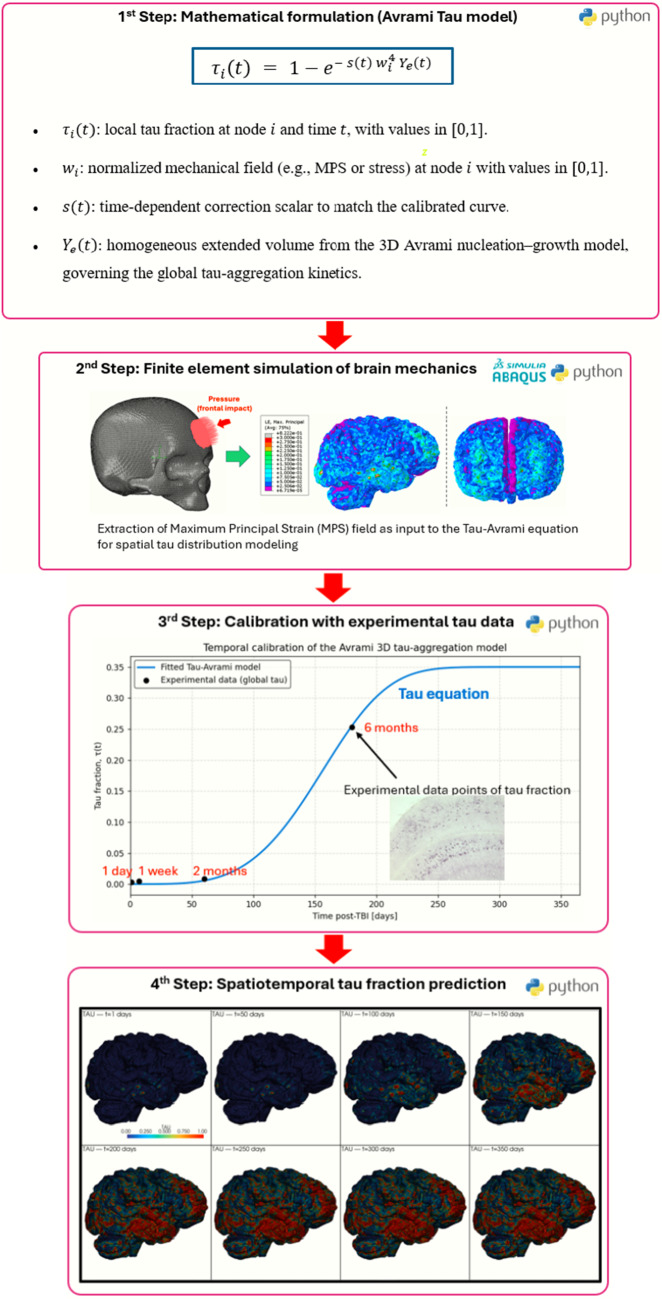
Workflow of the spatiotemporal modelling framework for tau aggregation following TBI

The resulting integration of calibrated temporal kinetics with the strain-derived spatial field produced a spatiotemporal distribution of tau accumulation across the virtual brain model. The overall methodological workflow is summarized in Fig. 1.

### Experimental dataset

Temporal calibration was based on four post-injury measurements of AT8-positive tau burden, expressed as AT%, which is the percentage of the analyzed histological area occupied by pathological tau detected by the AT8 monoclonal antibody, specifically recognizing abnormally phosphorylated tau. The four AT% values were obtained at 1,7,60, and 180 days post-injury, with representative mean burdens of 0.0032%, 0.0048%, 0.0085%, and 0.2533%, respectively. These measurements summarize the global progression of tau pathology reported in tau-transgenic mice subjected to moderate–severe TBI in Edwards et al. (2020). In that study, the injury consisted of a single CCI delivered after craniotomy directly onto the exposed cortex in one cerebral hemisphere, which can be mechanically interpreted as a localized indentation-type insult. Each value corresponds to the mean AT% averaged across cortex, hippocampus, and brainstem, providing a single global measure of pathological tau burden at each time point. It is important to note that these experimentally observed AT% values remain extremely low, reaching only ~ 0.25 AT% at six months, which reflects the very limited proportion of tissue occupied by AT8-positive aggregates in this model. Doing so, the dataset defines a low-magnitude global AT% trajectory, which was used directly as the calibration target for the Avrami kinetic parameters.

### Mathematical modelling of tau aggregation

The spatiotemporal evolution of misfolded tau aggregates is modeled using a Kolmogorov–Johnson–Mehl–Avrami (KJMA) nucleation–growth framework in a deterministic manner. In a stochastic formulation, quantities such as nucleation events and growth rates are treated as random variables, and different realizations of the model can produce different tau-aggregation trajectories even under the same conditions. In contrast, a deterministic formulation assigns a unique, reproducible evolution of the transformed % once the parameters are fixed. Because the objective here is to describe the averaged evolution of tau burden at the tissue scale and to match a single calibrated tau-kinetics curve exactly, a deterministic KJMA model is adopted. This class of models is widely used to represent phase transformations driven by the appearance of new domains and their subsequent growth, and it reproduces sigmoidal, saturation-type kinetics commonly observed in aggregation processes. Tau pathology progression exhibits these same characteristics: new misfolded “seeds” arise over time and existing aggregates expand, producing global kinetics for which an Avrami-type law provides a natural and analytically tractable description.

In quantitative histopathology, tau burden is assessed from AT8-immunostained sections as the percentage of tissue area showing AT8-positive labeling. In the present work, all quantities are therefore interpreted explicitly as the percentage of tau in the region or node under consideration.

To formalize this biological process mathematically, several assumptions are introduced. Misfolded tau seeds are idealized as nuclei that emerge deterministically according to a time-dependent nucleation rate. Each nucleus then grows radially with a deterministic velocity that varies exponentially in time, accommodating both accelerating and decelerating propagation regimes. Nuclei are considered perfectly spherical, which yields closed-form expressions for the extended and true transformed %. These assumptions enable transparent calibration against experimental tau-kinetics data and facilitate subsequent coupling to biomechanical fields.

Two temporal variables are employed. The observation time $t$ denotes the instant at which the transformed % is evaluated, whereas the variable $t^{\prime}$ denotes the birth time of an individual nucleus. This variable spans all nucleation events from the initial time $t=0$ up to the observation time. At each instant $t^{\prime}$, new nuclei appear at a nucleation rate

1 $$J({t}^{^{\prime}})={J}_{0}\hspace{0.17em}{e}^{{k}_{J}{t}^{^{\prime}}},$$

where ${J}_{0}$ is a reference nucleation rate (number of nuclei per unit volume and unit time) and ${k}_{J}$ controls temporal changes in nucleation. A positive ${k}_{J}$ represents increasing nucleation, while a negative ${k}_{J}$ decreasing nucleation; ${k}_{J}=0$ corresponds to constant nucleation.

Following a nucleation at time ${t}^{^{\prime}}$, each nucleus grows radially with velocity

2 $$v(t)={v}_{0}\hspace{0.17em}{e}^{{k}_{v}t},$$

where ${v}_{0}$ is a reference growth velocity and ${k}_{v}$ determines whether growth accelerates, decelerates, or remains constant. The radius at time $t$ of a nucleus born at ${t}^{^{\prime}}$ is obtained by integrating the instantaneous velocity from ${t}^{^{\prime}}$ to $t$. The integration variable of time is denoted by $s$:

3 $$R(t,{t}^{^{\prime}})={\int}_{{t}^{^{\prime}}}^{t}v(s)\hspace{0.17em}ds={\int}_{{t}^{^{\prime}}}^{t}{v}_{0}\hspace{0.17em}{e}^{{k}_{v}s}\hspace{0.17em}ds=\frac{{v}_{0}}{{k}_{v}}({e}^{{k}_{v}t}-{e}^{{k}_{v}{t}^{^{\prime}}}).$$

Under the assumption of spherical growth, the volume of this domain at time $t$ is

4 $$V(t,{t}^{^{\prime}})=\frac{4\pi }{3}\hspace{0.17em}{R}^{3}(t,{t}^{^{\prime}}).$$

The extended volume ${Y}_{e}(t)$ is defined as the total volume that all nuclei would transform if they did not overlap. The infinitesimal volume contribution from nuclei formed between ${t}^{^{\prime}}$ and ${t}^{^{\prime}}+d{t}^{^{\prime}}$ is:

5 $$d{Y}_{e}=J({t}^{^{\prime}})\hspace{0.17em}V(t,{t}^{^{\prime}})\hspace{0.17em}d{t}^{^{\prime}},$$

and therefore

6 $${Y}_{e}(t)={\int}_{0}^{t}J({t}^{^{\prime}})\hspace{0.17em}\frac{4\pi }{3}\hspace{0.17em}{R}^{3}(t,{t}^{^{\prime}})\hspace{0.17em}d{t}^{^{\prime}}.$$

Substituting the explicit forms of $J({t}^{^{\prime}})$ and $R\left(t,{t}^{^{\prime}}\right)$ yields

7 $$ Y_{e} \left( t \right) = \frac{{4\pi }}{3}J_{0} \left( {\frac{{v_{0} }}{{k_{v} }}} \right)^{3} \int\limits_{0}^{t} {e^{{k_{J} t^{\prime}}} } \left( {e^{{k_{v} t}}  - e^{{k_{v} t^{\prime}}} } \right)^{3} dt^{\prime}, $$

which evaluates to the closed-form expression

8 $$ Y_{e} (t) = \frac{{4\pi }}{3}J_{0} \left( {\frac{{v_{0} }}{{k_{v} }}} \right)^{3} \left[ {\frac{{e^{{(k_{J}  + 3k_{v} )t}}  - e^{{3k_{v} t}} }}{{k_{J} }} - 3\frac{{e^{{(k_{J}  + 3k_{v} )t}}  - e^{{2k_{v} t}} }}{{k_{J}  + k_{v} }} + 3\frac{{e^{{(k_{J}  + 3k_{v} )t}}  - e^{{k_{v} t}} }}{{k_{J}  + 2k_{v} }} - \frac{{e^{{(k_{J}  + 3k_{v} )t}}  - 1}}{{k_{J}  + 3k_{v} }}} \right]. $$

To incorporate overlap among growing domains, the KJMA formulation defines the true homogeneous transformed % as

9 $$X\left(t\right)=1-{e}^{-{Y}_{e}\left(t\right)}.$$

The notation $X(t)$ is exclusively reserved for this true homogeneous transformed percentage %, while ${Y}_{e}(t)$ denotes the corresponding extended volume.

However, allowing $X(t)$ to grow unrestrictedly would correspond to an almost complete replacement of tissue by pathological tau at the macroscopic scale, which is biologically unrealistic. In the experimental dataset used for calibration, the global AT8-positive burden across cerebral cortex, hippocampus and brainstem in tau-transgenic mice subjected to TBI remains extremely low, reaching only ≈0.25 AT% at 180 days post-injury (Iii et al. 2020). Consistent with these very small experimental values and the low burdens reported in the literature (Giannini et al. 2021), an upper bound $\alpha$ of 0.35% tau is introduced for the homogeneous model and defined as

10 $$X\left(t\right)=\alpha \left(1-{e}^{-{Y}_{e}\left(t\right)}\right)=0.35\left(1-{e}^{-{Y}_{e}\left(t\right)}\right).$$

In this way, $X\left(t\right)$ saturates at *α* % rather than approaching unrealistically high values. For the specific case considered here, $\alpha$ is set to 0.35%, which lies slightly above the six-month murine value (τ ≈ 0.25 AT%) reported by Edwards et al. (Iii et al. 2020), keeps the global tau burden within the sub-percent % range compatible with current quantitative histological observations in early and moderate tauopathy, and avoids biologically implausible near-complete replacement of brain parenchyma by tau aggregates (McKee et al. 2009; McKee et al. 2013). Importantly, the numerical value $\alpha$ of 0.35% is not fixed by the structure of the model: it can be straightforwardly modified in future applications if new calibration datasets become available that report higher long-term % burdens (for example, in advanced CTE or other late-stage tauopathies), without altering the underlying KJMA nucleation–growth formulation.

#### Coupling to the finite-element mechanical field

The kinetic model is afterwards integrated within a FE representation of brain biomechanics (Carmo et al. 2023). Each node $i$ of the mesh carries a scalar mechanical quantity ${w}_{0,i}$ (for example, MPS, stress magnitude, or strain–energy density). Since this field has arbitrary units and range, it is normalized to the interval $[\mathrm{0,1}]$ as

11 $${w}_{i}=\frac{{w}_{0,i}-\mathrm{m}\mathrm{i}\mathrm{n}({w}_{0})}{\mathrm{m}\mathrm{a}\mathrm{x}({w}_{0})-\mathrm{m}\mathrm{i}\mathrm{n}({w}_{0})},$$

where $\mathrm{m}\mathrm{i}\mathrm{n}({w}_{0})$ and $\mathrm{m}\mathrm{a}\mathrm{x}({w}_{0})$ denote the minimum and maximum values in the mesh. The normalized value ${w}_{i}$ is thus dimensionless, with ${w}_{i}=0$ corresponding to minimal mechanical stimulus and ${w}_{i}=1$ to maximal stimulus.

A nonlinear sensitivity of tau aggregation to mechanical loading is represented by the factor ${w}_{i}^{4}$, which amplifies differences between regions with high and low mechanical stimulus so that nodes with relatively large ${w}_{i}$ will contribute significantly more to tau aggregation than nodes with small ${w}_{i}$. This choice enhances spatial contrast in the predicted tau distribution and yields a more focal pattern than quadratic or cubic mappings, which would preserve a greater contribution from intermediate values of ${w}_{i}$ and therefore produce smoother redistributions. However, this exponent is a modeling assumption adopted for this exploratory framework, not a validated mechanobiological law, and may be revised when spatially resolved tau data become available. Using this weighting, a local extended variable is defined at each node as

12 $${Y}_{i}(t)={w}_{i}^{4}\hspace{0.17em}{Y}_{e}(t).$$

A direct Avrami-type definition of the local transformed percentage % would be

13 $${\tau}_{i}(t)=1-{e}^{-{Y}_{i}(t)}.$$

However, this expression does not guarantee that the spatial average coincides with the calibrated homogeneous curve $X(t)=0.35 \left(1-{e}^{-{Y}_{e}(t)}\right)$. The average would be

14 $$\frac{1}{N}\sum_{i=1}^{N}(1-{e}^{-{w}_{i}^{4}{Y}_{e}(t)}),$$

which for a general distribution of ${w}_{i}$ differs from $1-{e}^{-{Y}_{e}(t)}$. Equality for all $t$ occurs only in the trivial case ${w}_{i}=1$ for every node. Without correction, the global average of the heterogeneous model cannot reproduce the calibrated homogeneous Avrami kinetics.

To enforce exact agreement, a deterministic, time-dependent scalar $S(t)$ is introduced, and the local % is defined as:

15 $${\tau}_{i}(t)=1-{e}^{-\hspace{0.17em}S(t)\hspace{0.17em}{Y}_{i}(t)}=1-{e}^{-\hspace{0.17em}S(t)\hspace{0.17em}{w}_{i}^{4}{Y}_{e}(t)}.$$

The corrective factor $S(t)$ is chosen such that

16 $$\frac{1}{N}\sum_{i=1}^{N}(1-{e}^{-\hspace{0.17em}S(t)\hspace{0.17em}{w}_{i}^{4}{Y}_{e}(t)})=0.35 \left(1-{e}^{-{Y}_{e}(t)}\right)=X(t),$$

ensuring that the spatially averaged kinetics match the calibrated homogeneous curve exactly while preserving the spatial distribution dictated by the mechanical field.

#### Optional incorporation of an impact amplitude factor ${\boldsymbol{A}}$

In addition to the normalized mechanical field, it is possible to introduce a global impact-amplitude factor $A$ defined as

17 $$A=\left(\frac{Q}{{Q}_{\mathrm{r}\mathrm{e}\mathrm{f}}}\right)\left(\frac{N}{{N}_{\mathrm{r}\mathrm{e}\mathrm{f}}}\right),$$

where $Q$ is the impact magnitude, $N$ is the number of impacts or loading cycles, and ${Q}_{\mathrm{r}\mathrm{e}\mathrm{f}}$, ${N}_{\mathrm{r}\mathrm{e}\mathrm{f}}$ are reference values. In this case, the local extended variable becomes

18 $${Y}_{i}(t)={A}^{4}\hspace{0.17em}{w}_{i}^{4}\hspace{0.17em}{Y}_{e}(t),$$

and the local transformed % reads

19 $${\tau}_{i}(t)=1-{e}^{-\hspace{0.17em}S(t)\hspace{0.17em}{A}^{4}{w}_{i}^{4}{Y}_{e}(t)}.$$

The factor $A$ thus may provide a compact way to encode global variations in the impact magnitude and the number of impacts, which are not captured by the normalized field ${w}_{i}$. In the present work, the calibration of tau kinetics is performed on the homogeneous curve $X(t)$ and the mechanical field is normalized, so $A$ is set to $1$, so impact mechanics affect only the spatial distribution of tau through ${w}_{i}^{4}$.

### Sensitivity analysis

To assess the influence of the kinetic parameters on the temporal evolution of tau, a one-factor-at-a-time sensitivity analysis is performed on the homogeneous Avrami model. In this analysis, the spatial field was not used; instead, the derived tau % transformed $\tau (t)$ is evaluated. Four parameters were considered: the initial radial growth velocity ${v}_{0}$, the initial nucleation rate ${J}_{0}$, and their temporal modulation coefficients ${k}_{v}$ and ${k}_{J}$. A baseline value of ${v}_{0}={J}_{0}={k}_{v}={k}_{J}={10}^{-3}$ was defined, and each parameter was independently perturbed by multiplicative factors $f\in \{\mathrm{0.5,0.8,1.0,1.2,1.5}\}$, while the remaining parameters remained fixed at baseline. For each perturbation, the corresponding trajectory $\tau (t)$ was computed analytically, allowing direct comparison of the effects on onset time, curve steepness, and progression toward the prescribed saturation level of $\tau =0.35$.

### Material modelling

The soft brain tissues were modeled as visco-hyperelastic materials, in which the constitutive response combines an instantaneous hyperelastic component with a time-dependent viscoelastic contribution.

The instantaneous response was described using a Neo-Hookean strain–energy density function written in compressible form for numerical implementation, while the material parameters were selected to approximate the nearly incompressible behavior characteristic of soft brain tissue.

For a deformation gradient tensor $\mathbf{F}$, with right Cauchy–Green tensor $\mathbf{C}={\mathbf{F}}^{\mathrm{T}}\mathbf{F}$, first invariant ${I}_{1}=tr(\mathbf{C})$, and elastic volume ratio ${J}_{\mathrm{e}\mathrm{l}}=\mathrm{d}\mathrm{e}\mathrm{t}(\mathbf{F})$, the strain-energy function was defined as

20 $$W={C}_{1}({\stackrel{`}{I}}_{1}-3)+\frac{1}{{D}_{1}}{({J}_{\mathrm{e}\mathrm{l}}-1)}^{2},$$

where ${\stackrel{`}{I}}_{1}={J}_{\mathrm{e}\mathrm{l}}^{-2/3}{I}_{1}$ is the isochoric part of the first invariant. The material constants ${C}_{1}$ and ${D}_{1}$ are related to the instantaneous shear modulus ${\mu}_{0}$ and bulk modulus $\kappa$ through

21 $${C}_{1}=\frac{{\mu}_{0}}{2},{D}_{1}=\frac{2}{\kappa }.$$

Assuming near-incompressibility, $\kappa$ was chosen several orders of magnitude larger than ${\mu}_{0}$, following standard practice in soft-tissue modelling.

To capture time-dependent relaxation, the hyperelastic response was supplemented by a quasi-linear viscoelastic (QLV) formulation. The stress at any time $t$ was computed as the convolution of the elastic response with a reduced relaxation function $g(t)$. The latter was represented as a finite Prony-series expansion,

22 $$g(t)={g}_{\infty }+\sum_{i=1}^{N}{g}_{i}\hspace{0.17em}{e}^{-t/{\tau}_{i}},$$

where ${g}_{\infty }$ is the long-term modulus fraction, ${g}_{i}$ is the contribution of the $i$-th Maxwell branch, and ${\tau}_{i}$ is the corresponding relaxation time. This formulation ensures that the instantaneous behaviour matches the Neo-Hookean response, while the exponential decay terms govern the temporal attenuation of stresses under constant strain. The resulting visco-hyperelastic model provides the nonlinear, rate-dependent mechanical behaviour characteristic of brain tissue under dynamic loading conditions.

## Results

### Sensitivity analysis

**Fig. 2:**
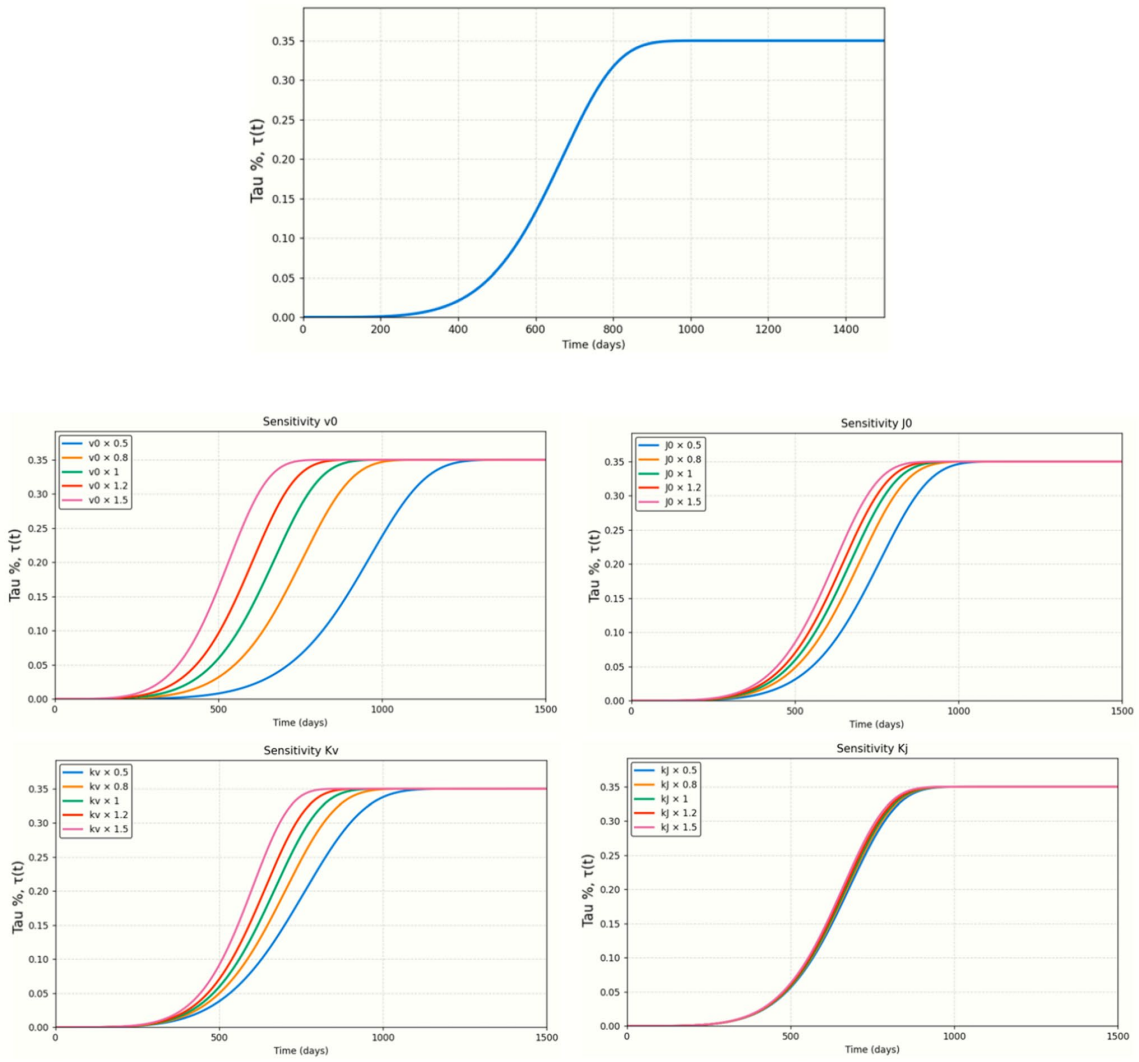
Sensitivity analysis of the Tau-Avrami kinetic parameters ${v}_{0}, {J}_{0}, {k}_{v}, {k}_{J}$

Figure 2 shows the effect of varying each kinetic parameter of the Avrami model around the baseline values ${v}_{0}={J}_{0}={k}_{v}={k}_{J}={10}^{-3}$. Each parameter was perturbed independently by multiplicative factors (0.5–1.5) while keeping the others fixed. Changes in ${v}_{0}$ and ${k}_{v}$ produce the most significant shifts, advancing or delaying the sigmoidal rise of $\tau (t)$. These parameters control the radial growth of tau-affected domains; increasing them accelerates the entire trajectory, while decreasing them slows it down. Variations in ${J}_{0}$ mainly affect the onset of the curve: higher nucleation rates produce an earlier rise in τ, whereas lower values postpone the initial increase. Finally, ${k}_{J}$ shows a comparatively smaller influence within the tested range, producing only modest horizontal shifts in the curve. Overall, all perturbations preserve the characteristic sigmoidal form, confirming the structural stability of the model while highlighting that growth-related parameters (${v}_{0}$, ${k}_{v}$) and baseline nucleation (${J}_{0}$) are the dominant drivers of temporal dynamics.

### Calibration

The kinetic parameters of the homogeneous Avrami model were calibrated to reproduce four experimental measurements of global tau burden at 1, 7, 60, and 180 days after TBI (τ = 0.0032, 0.0048, 0.0085, and 0.2533, respectively). These values correspond to the mean tau % across the cerebral cortex, hippocampus, and brainstem (Iii et al. 2020).

The four unknown kinetic parameters $({v}_{0},{J}_{0},{k}_{v},{k}_{J})$ were determined by minimizing the discrepancy between model predictions and experimental measurements. A two-step numerical procedure was implemented: (1) a broad random exploration of parameter space using NumPy-generated samples to identify promising regions, followed by (2) local refinement through SciPy’s *minimize* function (Nelder–Mead method), which iteratively reduced the mean-squared error between predictions and observations.

**Fig. 3:**
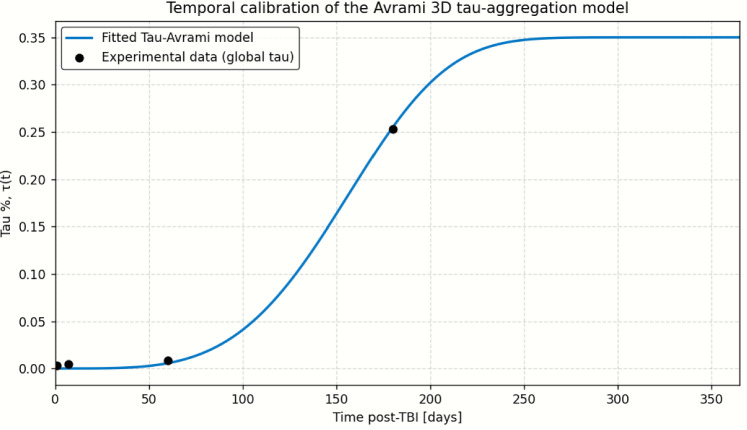
Calibration of the Tau-Avrami model against experimental tau measurements over time

Figure 3 shows the calibrated Avrami curve over the interval 0–365 days, together with the four experimental measurements used for calibration.

The resulting trajectory exhibits the expected dynamics: a nearly quiescent first week, modest accumulation by two months, and a strong nonlinear increase approaching 180 days. This calibrated time course (Fig. 3) serves as the global reference curve for all subsequent spatially heterogeneous simulations.

The corresponding model predictions at the four calibration times are listed in Table 1, along with the absolute error, the global root-mean-squared error (RMSE = 5.230 $\times$ 10^–4^), and the coefficient of determination (R^2^ = 0.9998). The calibrated curve reproduces values almost exactly.

**Table 1:** Experimental and model-predicted tau% at the calibration time points

Time (days)	τ (experimental)	τ (predicted)	|Δτ|	RMSE	${R}^{2}$
1.0	0.0032	0.00388	0.00068	5.230 $\times$ 10^−4^	0.99998
7.0	0.0048	0.00448	0.00032		
60.0	0.0085	0.00794	0.00056		
180.0	0.2533	0.25376	0.00046		

Calibrated values of the nucleation and growth parameters ${v}_{0}$, ${J}_{0}$, ${k}_{v}$, and ${k}_{J}$ obtained to match experimental tau-progression data are summarized in Table 2.

**Table 2:** Optimized kinetic parameters of the Avrami tau-aggregation model

Calibrated parameters			
${v}_{0}$[$mm/day$]	${J}_{0}$[nuclei mm^−1^ day ^−1^ ]	${k}_{v}$[day ^−1^]	${k}_{J}$[day ^−1^]
0.0472197	1.12609e-05	2.89573e-06	3.14002e-06

### Finite element analysis: brain biomechanics

**Fig. 4:**
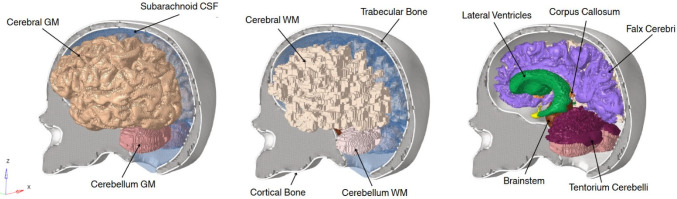
Anatomical components of the head model used for the finite element analysis (FEA)

A three-dimensional head model was reconstructed from a T1-weighted MRI using 3D Slicer. The segmentation included cerebral gray matter (GM), cerebral white matter (WM), cerebellar GM and WM, brainstem, corpus callosum, lateral ventricles, cerebrospinal fluid (CSF), falx cerebri, tentorium cerebelli, and cortical and trabecular bone. Surface imperfections and mesh artifacts were corrected using Autodesk Meshmixer, and the volumetric FE mesh was generated in Altair HyperMesh. The resulting geometry was topologically consistent and suitable for FE simulation. Figure 4 illustrates the anatomical components included in the model.

Bulk intracranial tissues were meshed using 8-node hexahedral elements with reduced integration (C3D8R). The final mesh contained 1,507,255 elements, including 1,399,893 solid elements and 107,362 shell elements corresponding to the dura mater and tentorium. The brain components presented an average element size of approximately 1 ${\mathrm{m}\mathrm{m}}^{3}$. Mesh quality was assessed using standard element-quality indices, including Jacobian, warpage, aspect ratio, minimum angle, and maximum angle. Overall, the mesh showed acceptable quality for dynamic finite-element simulation: for a Jacobian threshold of 0.5, all brain components exceeded 98% of elements passing the criterion, with minimum Jacobian values consistently around 0.45 or higher. Cerebral GM and WM, cerebellar GM and WM, brainstem, CSF, and lateral ventricles presented near-excellent values, while the lowest Jacobian percentage was observed in cortical bone. Additional quality metrics also showed generally satisfactory values, although some localized limitations were observed for warpage and maximum-angle criteria in small or geometrically complex structures such as the pituitary gland and trabecular bone.

The constitutive parameters summarized in Table 3 were adopted from previously published studies following the characterization strategy used in the female finite element head model (FeFEHM) of Carmo et al. (2023). Specifically, the viscoelastic parameters assigned to cerebral and cerebellar GM and WM, brainstem, and corpus callosum were based on the magnetic resonance elastography-informed characterization reported by Alshareef et al. (2021; 2025), while the Neo-Hookean hyperelastic parameters of the soft brain tissues were supported by the regional microindentation data of Menichetti et al. (2020). The constitutive parameters of the CSF and lateral ventricles were taken from Gilchrist (2003) using a Mooney–Rivlin formulation. The pituitary gland properties were adopted from Bouchonville et al. (2016), the meningeal structures (falx, tentorium, and dura mater) were assigned linear elastic properties according to Galford and McElhaney (1970), and the skull material characterization was based on Barbosa et al. (2020) for cortical and trabecular bone. Accordingly, Table 3 includes visco-hyperelastic properties for the soft brain tissues, hyperelastic properties for CSF/lateral ventricles, linear elastic properties for the pituitary gland and meningeal structures, and quasi-brittle and elastic–plastic descriptions for the skull tissues.

**Table 3:** Material model parameters of the brain components

Component	Formulation	Parameters							
Cerebral GM	Visco-hyperelastic	1045	8.32	0.654	0.101	0.067	0.6	7.5	60.7
Cerebral WM		1041	9.82	0.654	0.129	–	1.0	21.5	–
Cerebellum GM		1045	9.80	0.752	0.084	0.049	0.3	5.8	53.3
Cerebellum WM		1045	7.48	0.647	0.137	–	0.7	16.1	–
Brainstem		1045	6.66	0.549	0.126	0.085	0.6	7.8	39.5
Corpus callossum		1041	4.49	0.570	0.220	–	21.0	300.0	–
CSF/lateral ventricles	Hyperelastic	ρ [kg/m^3^]		C10 [MPa]		C01 [MPa]		D1 [MPa-1]	
		1000		0.9		1		0.9	
		ρ [kg/m^3^]		Young’s modulus[MPa]		Poisson’s ratio			
Pituitary gland	Linear elastic	1041		9.5 × 10⁻^3^		ν = 0.45			
Falx/tentorium/dura mater		1130		31.5		ν = 0.45			
Cortical bone	Quasi-brittle	1900		20,000		ν = 0.21			
Trabecular bone	Elastic–plastic	1500		1000		ν = 0.05			

To emulate a mild-to-moderate traumatic brain impact, a short-duration pressure pulse was applied over a frontal region of the skull, approximately normal to the forehead surface. This loading condition was selected as a biomechanically plausible frontal-impact scenario within a previously validated head-modeling framework. In particular, the adopted head-modeling strategy follows the FeFEHM framework of Carmo et al. (2023), while the use of a frontal impact configuration is consistent with classical frontal head-impact validation studies by Nahum et al. (1977) and subsequent numerical assessments of the same modeling strategy by (Gomes et al. 2024). In the present study, its role is to generate a meaningful intracranial strain field for coupling with the tau aggregation model.

The load rose sharply and decayed within a total simulated time of 14 ms, capturing the rapid mechanical insult characteristic of a frontal head impact. The head model was unconstrained, since the brief 14 ms pressure pulse duration did not produce unrealistic rigid-body motion while still permitting realistic intracranial deformation. An unconstrained configuration was preferred to avoid artificial stress concentrations associated with fixed supports, particularly near inferior regions such as the brainstem, and to allow the propagation of strain waves throughout the brain during the short-duration dynamic event.

**Fig. 5:**
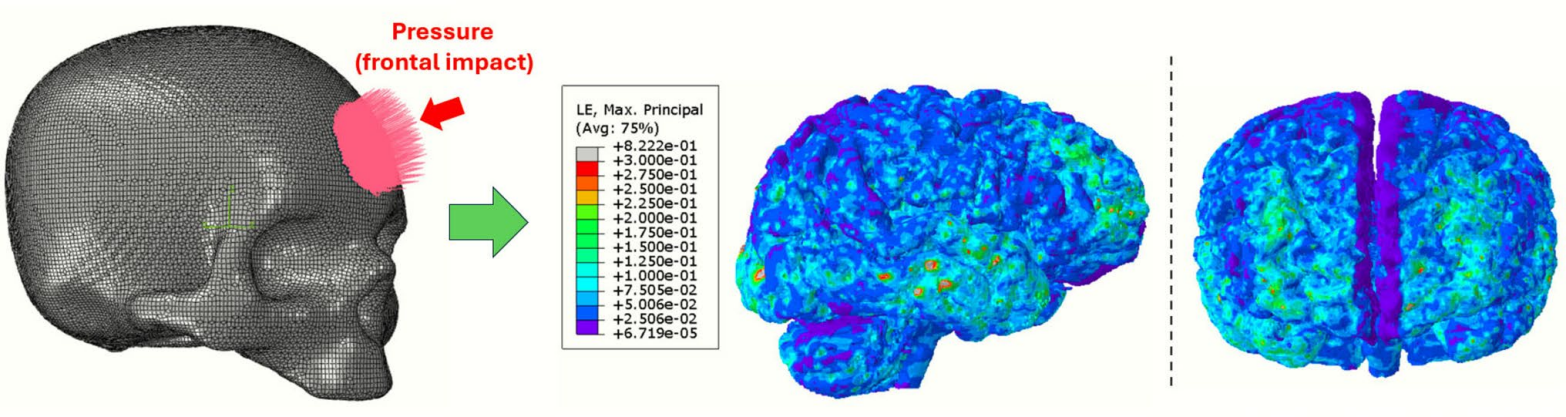
FE impact simulation: boundary conditions, applied frontal pressure, and resulting MPS field

From the simulated response, the MPS was computed throughout all brain tissues. The MPS value at the final simulation time (*t* = 0.014 s) was extracted at each node and used as the mechanical field $w(x)$ in the tau-aggregation model. As shown in Fig. 5, elevated MPS values localized predominantly in frontal cortical regions, forming the basis for the spatial weighting of tau progression. After normalization, this field was passed to the Python implementation of the Avrami kinetics, such that regions experiencing greater mechanical strain subsequently develop higher tau percentages over time, providing a direct mechanistic link between acute TBI biomechanics and subsequent spatial progression of tau pathology considered in this study.

### Spatiotemporal tau maps and atrophy surrogate

**Fig. 6:**
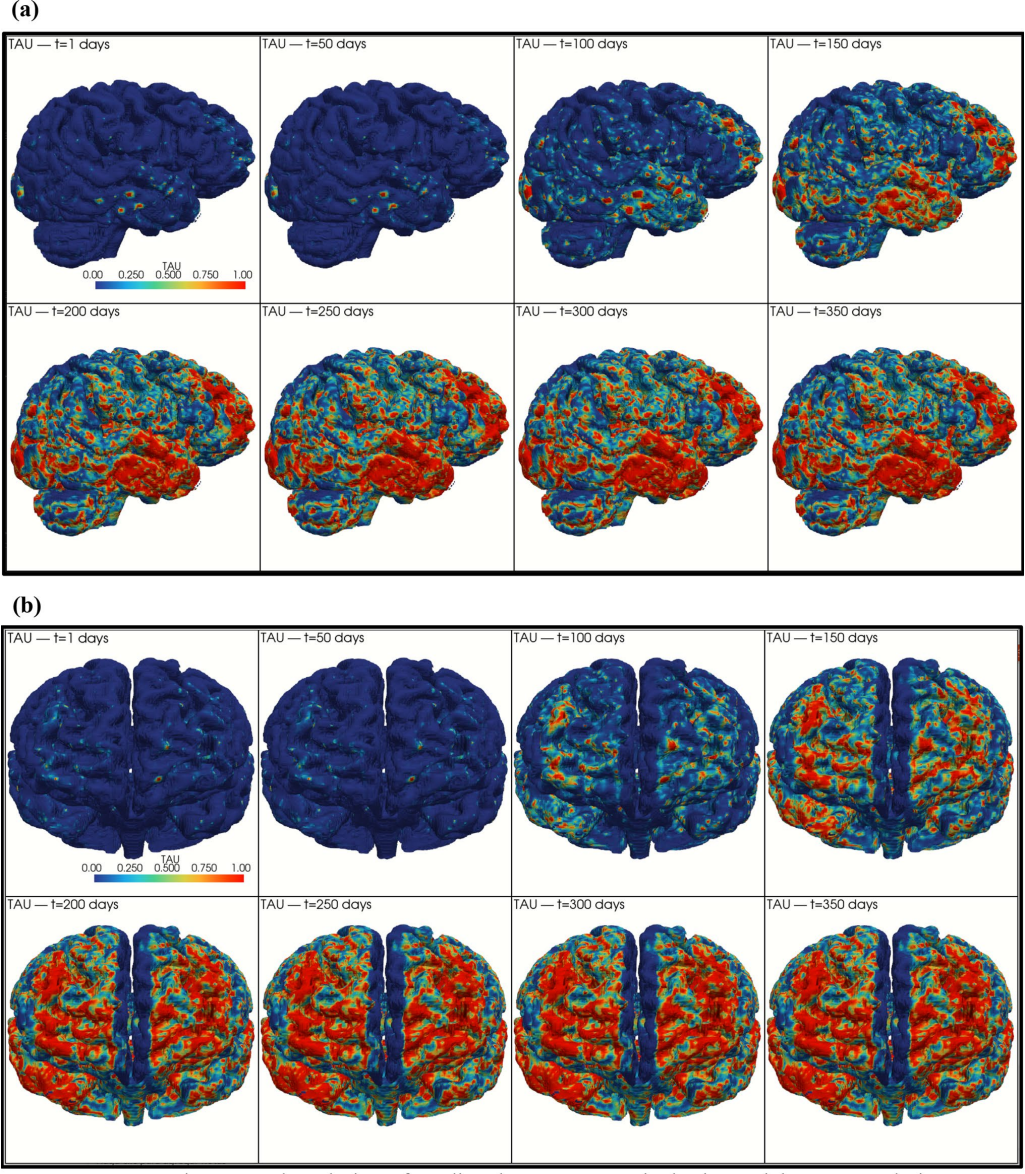
Spatiotemporal evolution of predicted tau % across the brain model. **a** Lateral view. **b** Frontal view

The integration of the calibrated temporal law with the MPS-derived mechanical field generated spatiotemporal maps $\tau (x,t)$, describing how tau burden distributes across the brain over time. Figure 6 illustrates this evolution using eight time points (1, 50, 100, 150, 200, 250, 300, and 350 days), shown from a lateral view (Fig. 6a) and a frontal view (Fig. 6b).

At the earliest stages (1–50 days), tau levels remain extremely low and scattered, reflecting the near-zero values predicted by the calibrated global curve. Between 100 and 150 days, localized regions of higher $\tau$ begin to emerge, predominantly in areas that experienced elevated MPS during the initial impact. These clusters demonstrate that spatial vulnerability is inherited directly from the mechanical loading pattern.

Between 200 and 250 days, tau accumulation becomes more widespread, and isolated clusters merge into larger, contiguous regions. Importantly, although the spatial maps show increasing regional involvement, the global average tau % across all nodes follows the calibrated temporal trajectory and begins to stabilize after approximately 250 days. Consequently, the maps from 250 to 350 days exhibit equal results.

This behavior highlights a key feature of the model in which mechanics determine where tau accumulates, while the calibrated kinetics determine how much tau accumulates over time.

**Fig. 7:**
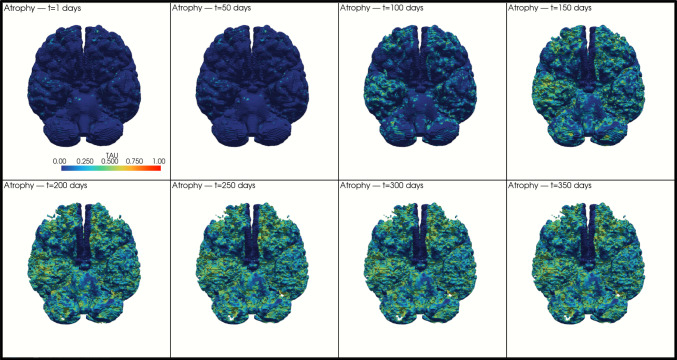
Spatiotemporal evolution of atrophy across the brain model

To illustrate the potential long-term structural consequences of tau accumulation, a simple atrophy surrogate was implemented in which mesh elements exceeding a local threshold of ${\tau}_{i}\ge 0.7$ % were removed. Although the global homogeneous model is constrained to a maximum of 0.35% tau burden, the local τᵢ values represent node-level percentages within the heterogeneous spatial field, allowing individual nodes to reach up to 1% as a theoretical upper bound of localized transformation. This removal generated hollow regions resembling focal tissue loss (Fig. 7). While conceptual, this approach suggests that atrophy would preferentially emerge in areas with high tau burden (originating from mechanically vulnerable regions) and that progressive tissue loss would reduce overall brain volume over time. If incorporated into the kinetics, such volumetric reduction would ultimately alter the shape of the global tau trajectory by decreasing the available tissue for further transformation, reinforcing a potential mechanistic continuum from mechanical injury to tau accumulation and structural degeneration.

## Discussion

The present study introduces a biomechanically informed Avrami-type formulation to describe the spatiotemporal progression of tau pathology following TBI, offering a conceptual and methodological advance over existing computational approaches. Traditional models of tau propagation, such as pure diffusion or connectivity-based frameworks (Cornblath et al. 2021; Zheng et al. 2019; Raj et al. 2012), or prion-like templating models that describe self-propagating protein aggregates (Jucker et al. 2013), have reproduced aspects of regional spreading; however, they generally lack a principled description of where and when pathological domains first emerge. In these formulations, pathology is often assumed to be already present and merely transported across structural or functional pathways, which limits their capacity to explain the heterogeneity in early focal deposition consistently reported in post-mortem studies of contact-sport athletes and individuals with repetitive head trauma (Butler et al. 2025). By contrast, the approach developed here explicitly separates nucleation from growth, grounding both in parameters modulated by the mechanical environment. Such a framework allows the impact-induced maximum principal strain field, well-documented as a trigger of axonal perturbation and injury-related tau aggregation (Bain and Meaney 2000; Meaney and Smith 2011), to directly shape the early spatial pattern of tau burden, thereby linking impact biomechanics to biochemical kinetics in a manner that closes a key gap in the literature.

A major distinction of the present model lies in its adoption of a KJMA-based nucleation–growth formulation, which is long established in materials science for describing phase transitions (Fanfoni et al. 1998; Avrami 1939) but has seldom been applied to neurodegenerative kinetics. The Avrami equation provides parameters whose physical interpretations naturally map onto biological processes: nucleation rates that reflect the probability of injury-induced misfolding events, and growth velocities that capture the radial expansion of tau-affected microdomains. Existing computational approaches to protein aggregation and neurodegenerative disease spread often describe aggregate accumulation through phenomenological rules (Honson et al. 2008; Jucker et al. 2013; Crespo et al. 2016), with limited mechanistic interpretability, making cross-study comparisons and load-dependent predictions challenging. The formulation proposed here incorporates exponential time modulation in both nucleation and growth, offering flexibility to reproduce accelerating or decelerating regimes while retaining the analytical closed-form structure of the Avrami solution (Fanfoni et al. 1998; Avrami 1939; Shirzad and Viney 2023).

Another key contribution is the integration of FE-derived mechanical fields into the kinetic model. FE simulations of brain trauma have been instrumental in characterizing stress, strain, and strain-rate distributions during impact (Wright et al. 2013; Zhan et al. 2025), consistently revealing mechanically vulnerable regions such as sulcal depths and gray–white matter interfaces, which also appear early in CTE (McKee et al. 2009, 2013). However, biomechanical metrics have typically been used either as isolated injury predictors or as correlational biomarkers, without a formal mechanism linking them to biochemical transformation. Conversely, models of tau spreading rarely incorporate mechanical inputs, focusing instead on network topology, connectivity, or empirical propagation rates (Cornblath et al. 2021; Zheng et al. 2019; Raj et al. 2012). The present framework unifies these domains by allowing the FE-computed mechanical field to modulate the local extended volume of tau through a nonlinear curve, consistent with experimental and modeling evidence that tau misfolding and accumulation are highly sensitive to mechanical perturbation thresholds and injury severity (Horstemeyer et al. 2019; Butler et al. 2025). The fourth-power sensitivity enhances spatial contrast by amplifying differences between highly and mildly stressed regions, mirroring observations that subthreshold insults produce little biochemical response. At the same time, higher magnitude loads disproportionately accelerate tau pathology (Iii et al. 2020; Horstemeyer et al. 2019).

The introduction of the normalization factor S(t) is essential for reconciling spatial heterogeneity with global calibration. Heterogeneous transformation models often distort their macroscopic kinetics when region-specific modifiers are introduced. By enforcing that the spatial average matches the experimental tau-transgenic trajectory derived from TBI-induced tau aggregation and spreading (Iii et al. 2020), S(t) ensures that the calibrated murine time course remains the global temporal backbone while local progression is shaped by biomechanics. This structural feature parallels normalization strategies used in other biomechanical and materials-transformation contexts to maintain comparability across heterogeneous scenarios (Fanfoni et al. 1998; Avrami 1939; Barmak and Barmak 2010).

The calibrated curve reproduces the slow-onset, accelerated-growth trajectory observed in models of tauopathy subjected to moderate to severe TBI (Iii et al. 2020), characterized by minimal transformation during the first week, subtle accumulation by two months, and a sharp rise by six months. This pattern aligns with neuropathological observations, which demonstrate early microscopic tau alterations followed by nonlinear increases in aggregate burden in CTE and related tauopathies (Honson et al. 2008; Guo et al. 2017; McKee et al. 2009, 2013). Previous computational efforts have either failed to capture these dynamics or relied on arbitrary time constants lacking biological grounding. Through a combination of global search and local optimization, the present model achieves excellent accuracy (R^2^ ≈ 0.999), preserving mechanistic interpretability while accurately fitting experimental observations.

When projected onto the human FE mesh, the model generates spatial tau distributions that qualitatively reproduce known CTE staging, including early perivascular and sulcal-accentuated involvement, spreading across cortical territories, and eventual coalescence of pathological regions, in accordance with classical descriptions by McKee et al. (2009, 2013). The atrophy surrogate (τ ≥ 0.7) produces hollowed-out regions resembling gyral thinning and cavitation in advanced cases, establishing a preliminary link between biochemical burden and macroscopic degeneration. (Schäfer et al. 2024) proposed a biologically grounded tau–atrophy relationships within personalized multiphysics frameworks inferred from longitudinal imaging. In contrast, the present surrogate offers a simple mechanically interpretable way to visualize where tissue loss could emerge from strain-driven tau accumulation. It should therefore be understood as a spatial proof of concept, not as a direct one-to-one tau–atrophy law and could be refined in future work.

The model's translational potential is particularly noteworthy. Because the FE simulation is conducted on a realistic human geometry, the framework could support subject-specific evaluation of mechanical vulnerability, extending emerging paradigms in personalized concussion biomechanics (Carmo et al. 2023; Griffiths and Budday 2022).

In comparison to existing literature, the present approach fills a crucial methodological gap. Until now, biomechanics and biochemical tau kinetics have largely been studied separately, with FE models emphasizing stress and strain distributions (Bain and Meaney 2000; Meaney and Smith 2011; Giordano and Kleiven 2014; Wright et al. 2013; Zhan et al. 2025; Carmo et al. 2023; Griffiths and Budday 2022) and neurodegeneration models emphasizing network-mediated spread and prion-like propagation (Cornblath et al. 2021; Zheng et al. 2019; Raj et al. 2012; Jucker et al. 2013). Attempts to bridge these domains include the mechanical brain damage framework proposed by Horstemeyer et al. (Horstemeyer et al. 2019), which relates cumulative mechanical damage metrics to abnormal tau accumulation in National Football League players. However, this formulation remains largely phenomenological and does not explicitly incorporate nucleation-and-growth kinetics or closed-form transformation laws. The current framework instead establishes a mechanistic, transformation-based link between spatial mechanical damage and pathological evolution of tau.

Overall, this research introduces a coherent theoretical and computational structure that integrates TBI biomechanics with tau aggregation biology. By leveraging the analytical strengths of the Avrami formulation, the anatomical specificity of FE modeling, and a biologically calibrated temporal framework informed by TBI-induced tau aggregation experiments (Iii et al. 2020), the study provides a foundation for predictive modeling of CTE-related degeneration.

### Limitations of the study and future directions

In this work, the mechanical input of the model is reduced to a single scalar field $w(x)$, obtained from the normalized MPS. This choice was made because $w(x)$ provides a straightforward surrogate for axonal mechanical damage and offers a practical way to demonstrate how biomechanics can drive tau aggregation in the model. However, this simplification means that other mechanical factors are not taken into account. As a result, the model captures only one dimension of the mechanical response to trauma and does not account for the full complexity of brain tissue loading. In addition, the nonlinear mapping between MPS and tau aggregation, here represented through a fourth-power weighting, remains phenomenological. Although it was selected to enhance focal spatial contrast, its quantitative form should be reassessed once spatially resolved tau pathology data become available.

Another central limitation is that the model establishes a direct relationship between MPS and tau aggregation, without incorporating other biochemical, cellular, or molecular factors known to influence tau pathology. Neuroinflammation, microglial activation, kinase/phosphatase balance, mitochondrial dysfunction, neurovascular alterations, and prion-like seeding dynamics may also modulate tau spreading and regional vulnerability (Honson et al. 2008; Guo et al. 2017). Because these processes are not represented, the model captures only one component (mechanical damage) of a plausible multiscale and multiphysics biological response to trauma. Therefore, the biological calibration is temporal, whereas the mechanical field determines the internal spatial weighting within each region. This means that the finite-element field does not calibrate the mean tau burden; it redistributes a previously calibrated regional burden over space. Accordingly, the spatial maps should be interpreted as mechanically informed redistributions of a biologically calibrated tau load, rather than as directly validated predictions of local tau concentration.

In addition, the present work calibrates the tau kinetics at a global scale. This simplification neglects well-established regional differences in tau spreading. A more refined calibration and application of the kinetic law on a region-specific basis would be required to reproduce the heterogeneous evolution of tau pathology observed experimentally. Moreover, the experimental temporal calibration is based on a single-impact murine study, whereas CTE is primarily associated with repetitive head impacts. Repeated-impact formulations, potentially including cumulative or threshold-dependent activation of tau pathology, should therefore be incorporated in future developments.

Finally, the model assumes fixed temporal kinetics derived from murine data and applies them directly to a human-scale mesh, which introduces uncertainties associated with cross-species scaling. Region-specific and human longitudinal datasets would provide a stronger empirical basis for future calibration. Future work should address this limitation through explicit scaling strategies. In the context of a localized cortical insult (Iii et al. 2020), geometric descriptors such as cortical thickness may provide a more mechanically meaningful basis for mouse-to-human scaling than global quantities such as whole-brain mass or total brain volume. The latter differ by several orders of magnitude between species and would therefore yield excessively large scaling factors, leading to unrealistic human equivalents of the experimental indentation geometry. By contrast, cortical thickness is directly related to the local tissue layer engaged by the insult and is therefore more physically consistent for translating a focal cortical deformation problem such as that of Edwards et al. (Iii et al. 2020). In addition to anatomical scaling, future work could use a mechanically based scaling strategy, in which murine and human FE loading amplitudes are adjusted until comparable cortical response metrics, such as peak MPS, stress, or strain-energy density, reach similar values. This would define load equivalence from the resulting mechanical response.

A further important step will be the implementation of an indentation-type finite-element simulation in a mouse brain model, more closely matching the experimental conditions of Edwards et al. (Iii et al. 2020), in order to compare predicted and observed spatial tau distributions directly rather than using the murine data only for temporal calibration.

An additional practical limitation concerns translational validation in humans. Longitudinal spatiotemporal measurements of post-traumatic tau pathology in living human subjects remain scarce, especially in cohorts exposed to repetitive head impacts. If richer long-term datasets become available in the future, they could be used to refine the temporal laws, improve regional specificity, and evaluate the extent to which the present framework captures clinically meaningful patterns of post-traumatic tau evolution.

## Conclusion

This exploratory research presents a mechanistically informed framework that connects the mechanical effects of TBI with the subsequent development of tau pathology. By integrating Avrami nucleation–growth kinetics with FE strain fields, the model captures both the calibrated time course of tau accumulation and its preferential localization in mechanically strained regions. The atrophy surrogate further illustrates how sustained tau burden may translate into tissue loss, suggesting a potential pathway from acute deformation to long-term degeneration relevant to CTE. Although simplified, the framework provides a quantitative basis for linking injury biomechanics to the progression of neurodegenerative diseases. It provides a foundation for future extensions, including repeated impacts, anisotropic propagation, and subject-specific simulations.

## Data Availability

No datasets were generated or analysed during the current study.

## References

[CR1] Alshareef A et al (2021) Integrating material properties from magnetic resonance elastography into subject-specific computational models for the human brain. Brain Multiphys. 10.1016/j.brain.2021.100038 10.1016/j.brain.2021.100038 PMC10168673 37168236

[CR2] Alshareef A, Carass A, Lu YC, Mojumder J, Diano AM, Bailey OM, Okamoto RJ, Pham DL, Prince JL, v. Bayly P, Johnson CL (2025) Average biomechanical responses of the human brain grouped by age and sex. Ann Biomed Eng. 10.1007/s10439-025-03725-y 10.1007/s10439-025-03725-y PMC12075284 40205286

[CR3] Avrami M (1939) Kinetics of phase change. I general theory. J Chem Phys. 10.1063/1.1750380

[CR4] Bain AC, Meaney DF (2000) Tissue-level thresholds for axonal damage in an experimental model of central nervous system white matter injury. J Biomech Eng. 10.1115/1.1324667 10.1115/1.1324667 11192383

[CR5] Barbosa A, Fernandes FAO, de Sousa RJA, Ptak M, Wilhelm J (2020) Computational modeling of skull bone structures and simulation of skull fractures using the YEAHM head model. Biology Basel 9(9):1–18. 10.3390/biology9090267 10.3390/biology9090267 PMC7566004 32899779

[CR6] Barmak K, Barmak K (2010) A Commentary on: reaction kinetics in processes of nucleation and growth. Metall Mater Trans B. 10.1007/s11663-010-9421-1

[CR7] Bouchonville N, Meyer M, Gaude C, Gay E, Ratel D, Nicolas A (2016) AFM mapping of the elastic properties of brain tissue reveal kPa/µm gradients of rigidity. Soft Matter. 10.1039/C6SM00582A 10.1039/c6sm00582a 27377831

[CR8] Butler MLMD et al (2025) Repeated head trauma causes neuron loss and inflammation in young athletes. Nature. 10.1038/s41586-025-09534-6 10.1038/s41586-025-09534-6 PMC12589125 40963024

[CR9] de Calignon A et al (2012) Propagation of tau pathology in a model of early Alzheimer’s disease. Neuron. 10.1016/j.neuron.2011.11.033 10.1016/j.neuron.2011.11.033 PMC3292759 22365544

[CR10] Carmo GP, Dymek M, Ptak M, Alves-de-Sousa RJ, Fernandes FAO (2023) Development, validation and a case study: The female finite element head model (FeFEHM). Comput Methods Programs Biomed. 10.1016/j.cmpb.2023.107430 10.1016/j.cmpb.2023.107430 36827824

[CR11] Cornblath EJ et al (2021) Computational modeling of tau pathology spread reveals patterns of regional vulnerability and the impact of a genetic risk factor. Sci Adv. 10.1126/sciadv.abg6677 10.1126/sciadv.abg6677 PMC8189700 34108219

[CR12] Covassin T, Stearne D, Elbin IR (2008) Concussion history and postconcussion neurocognitive performance and symptoms in collegiate athletes. J Athl Train. 10.4085/1062-6050-43.2.119 10.4085/1062-6050-43.2.119 PMC2267331 18345335

[CR13] Crespo R, Villar-Alvarez E, Taboada P, Rocha FA, Damas AM, Martins PM (2016) What can the kinetics of amyloid fibril formation tell about off-pathway aggregation? *. J Biol Chem. 10.1074/jbc.M115.699348 10.1074/jbc.M115.699348 PMC4722475 26601940

[CR14] Fanfoni M, Tomellini M, Fanfoni M, Tomellini M (1998) The Johnson-Mehl- Avrami-Kohnogorov model: a brief review. Nuovo Cim D. 10.1007/BF03185527

[CR15] Giordano C, Kleiven S (2014) Evaluation of axonal strain as a predictor for mild traumatic brain injuries using finite element modeling. Stapp Car Crash J. 10.4271/2014-22-0002 10.4271/2014-22-0002 26192949

[CR16] Galford JE, McElhaney JH (1970) A viscoelastic study of scalp, brain, and dura. J Biomech 3(2):211–221. 10.1016/0021-9290(70)90007-2 5521539 10.1016/0021-9290(70)90007-2

[CR17] Gardner RC, Yaffe K (2015) Epidemiology of mild traumatic brain injury and neurodegenerative disease. Mol Cell Neurosci. 10.1016/j.mcn.2015.03.001 10.1016/j.mcn.2015.03.001 PMC4461453 25748121

[CR18] Giannini LA et al (2021) Mapping tau burden and neuronal loss in MAPT-associated frontotemporal lobar degeneration. Alzheimer’s Dementia 17(suppl):3. 10.1002/alz.054141

[CR19] Gilchrist MD (2003) Modelling and accident reconstruction of head impact injuries. Key Eng Mater 245–246:417–432

[CR20] Gomes MS, Carmo GP, Ptak M, Fernandes FAO, Alves-de-Sousa RJ (2024) Accuracy and efficiency of finite element head models: the role of finite element formulation and material laws. Int J Num Methods Biomed Eng. 10.1002/cnm.3851 10.1002/cnm.3851 39045773

[CR21] Griffiths E, Budday S (2022) Finite element modeling of traumatic brain injury: Areas of future interest. Curr Opin Biomed Eng. 10.1016/j.cobme.2022.100421

[CR22] Guo T, Noble W, Hanger DP, Guo T, Noble W, Hanger DP (2017) Roles of tau protein in health and disease. Acta Neuropathol. 10.1007/s00401-017-1707-9 10.1007/s00401-017-1707-9 PMC5390006 28386764

[CR23] Honson NS, Kuret J, Jesus Avila GP, Smith MA, Honson NS, Kuret J (2008) Tau aggregation and toxicity in Tauopathic neurodegenerative diseases. J Alzheimer’s Dis. 10.3233/JAD-2008-14409 10.3233/jad-2008-14409 PMC2748882 18688092

[CR24] Horstemeyer MF et al (2019) A mechanical brain damage framework used to model abnormal brain tau protein accumulations of national football league players. Ann Biomed Eng. 10.1007/s10439-019-02294-1 10.1007/s10439-019-02294-1 PMC6757135 31372858

[CR25] Edwards III GE, Zhao J, Dash PK, Soto C, Moreno-Gonzalez I (2020) Traumatic brain injury induces tau aggregation and spreading. J Neurotrauma. 10.1089/neu.2018.6348 10.1089/neu.2018.6348 PMC6921297 31317824

[CR26] Jucker M, Walker LC, Jucker M, Walker LC (2013) Self-propagation of pathogenic protein aggregates in neurodegenerative diseases. Nature. 10.1038/nature12481 10.1038/nature12481 PMC3963807 24005412

[CR27] McInnes K, Friesen CL, MacKenzie DE, Westwood DA, Boe SG (2017) Mild traumatic brain injury (mTBI) and chronic cognitive impairment: a scoping review. PLoS ONE. 10.1371/journal.pone.0174847 10.1371/journal.pone.0174847 PMC5388340 28399158

[CR28] McKee AC et al (2009) Chronic Traumatic Encephalopathy in athletes: progressive tauopathy after repetitive head injury. J Neuropathol Exp Neurol. 10.1097/NEN.0b013e3181a9d503 10.1097/NEN.0b013e3181a9d503 PMC2945234 19535999

[CR29] McKee AC et al (2013) The spectrum of disease in chronic traumatic encephalopathy. Brain. 10.1093/brain/aws307 10.1093/brain/aws307 PMC3624697 23208308

[CR30] Meaney DF, Smith DH (2011) Biomechanics of concussion. Clin Sports Med. 10.1016/j.csm.2010.08.009 10.1016/j.csm.2010.08.009 PMC3979340 21074079

[CR31] Menichetti A, MacManus DB, Gilchrist MD, Depreitere B, Sloten JV, Famaey N (2020) Regional characterization of the dynamic mechanical properties of human brain tissue by microindentation. Int J Eng Sci. 10.1016/j.ijengsci.2020.103355

[CR32] Nahum AM, Smith R, Ward CC (1977) Intracranial pressure dynamics during head impact. SAE Techn Paper. 10.4271/770922

[CR33] Puri IK, Li L (2010) Mathematical modeling for the pathogenesis of Alzheimer’s disease. PLoS ONE. 10.1371/journal.pone.0015176 10.1371/journal.pone.0015176 PMC3001872 21179474

[CR34] Raj A, Kuceyeski A, Weiner M (2012) A network diffusion model of disease progression in dementia. Neuron. 10.1016/j.neuron.2011.12.040 10.1016/j.neuron.2011.12.040 PMC3623298 22445347

[CR36] Schäfer A, Chaggar P, Thompson TB, Goriely A, Kuhl E (2021) Predicting brain atrophy from tau pathology: a summary of clinical findings and their translation into personalized models. Brain Multiphys. 10.1016/j.brain.2021.100039

[CR37] Shirzad K, Viney C (2023) A critical review on applications of the Avrami equation beyond materials science. J R Soc Interface. 10.1098/rsif.2023.0242 10.1098/rsif.2023.0242 PMC10282574 37340781

[CR38] Vogel JW et al (2020) Spread of pathological tau proteins through communicating neurons in human Alzheimer’s disease. Nat Commun. 10.1038/s41467-020-15701-2 10.1038/s41467-020-15701-2 PMC7251068 32457389

[CR39] Wright RM, Post A, Hoshizaki B, Ramesh KT (2013) A multiscale computational approach to estimating axonal damage under inertial loading of the head. J Neurotrauma. 10.1089/neu.2012.2418 10.1089/neu.2012.2418 22992118

[CR40] Wu JW et al (2016) Neuronal activity enhances tau propagation and tau pathology in vivo. Nat Neurosci. 10.1038/nn.4328 10.1038/nn.4328 PMC4961585 27322420

[CR41] Zhan X et al (2025) Local and global effects of inertial force components producing brain strain during head impacts. Comput Biol Med. 10.1016/j.compbiomed.2025.111248 10.1016/j.compbiomed.2025.111248 41161153

[CR42] Zheng Y-Q et al (2019) Local vulnerability and global connectivity jointly shape neurodegenerative disease propagation. PLoS Biol. 10.1371/journal.pbio.3000495 10.1371/journal.pbio.3000495 PMC6894889 31751329

